# Temporal variation in circulating GDF15 over 24 h in healthy young males

**DOI:** 10.14814/phy2.70672

**Published:** 2025-11-24

**Authors:** Dorte B. Zilstorff, Michael M. Richter, Jens Hannibal, Henrik L. Jørgensen, Henriette P. Sennels, Rune E. Kuhre, Christoffer Clemmensen, Nicolai J. Wewer Albrechtsen

**Affiliations:** ^1^ Department of Clinical Biochemistry Copenhagen University Hospital – Bispebjerg and Frederiksberg Hospital Copenhagen Denmark; ^2^ Department of Clinical Medicine, Faculty of Health and Medical Sciences University of Copenhagen Copenhagen Denmark; ^3^ Department of Clinical Biochemistry Copenhagen University Hospital – Hvidovre Hospital Hvidovre Denmark; ^4^ Obesity and Liver Pharmacology, Integrated Physiology Research Novo Nordisk A/S Måløv Denmark; ^5^ Novo Nordisk Foundation Center for Basic Metabolic Research, Faculty of Health and Medical Sciences University of Copenhagen Copenhagen Denmark; ^6^ Copenhagen Center for Translational Research Copenhagen University Hospital – Bispebjerg and Frederiksberg Hospital Copenhagen Denmark

**Keywords:** 24‐h rhythm, circadian rhythm, diurnal rhythm, GDF15, metabolism

## Abstract

The functions of Growth Differentiation Factor 15 (GDF15) include actions on metabolism, cell survival, immune response, inflammation, and inhibition of food intake. Temporal variations in circulating GDF15 over 24 h have been reported in two small cohorts: one during fasted conditions and one during an overfeeding regimen. Here, 22 healthy young men were studied over 24 h in a controlled setting approximating normal daily life with blood sampling every third hour. Plasma GDF15 concentrations were analyzed using cosinor rhythmometry and one‐way repeated measures ANOVA. In the full cohort, cosinor analysis did not show a statistically significant 24‐h rhythm of GDF15 (*p* = 0.0944), but the ANOVA revealed a significant modest effect of time on plasma GDF15 concentrations (*p* < 0.001). Exploratory post hoc cosinor analysis of a subgroup of 14 subjects with evening‐peaking profiles indicated modest rhythmic fluctuations (*p* = 0.0467), but the effect was small compared with the fluctuations of other metabolic hormones and plasma changes in GDF15 due to, for example, cancer and pregnancy. These findings do not provide definitive evidence for a 24‐h rhythm of GDF15, but post hoc results suggest that some individuals may exhibit modest 24‐h fluctuations. Larger, prospectively powered studies are required to confirm these observations and clarify their clinical significance.

## 1 INTRODUCTION

Growth differentiation factor 15 (GDF15) is a member of the transforming growth factor‐β (TGF‐β) family (Sigvardsen et al., 2025). The functions of GDF15 are pleiotropic, but include appetite regulation, and actions on metabolism, cell survival, immune response, and inflammation (Iglesias et al., 2023). GDF15 acts through the glial cell‐derived neurotrophic factor (GDNF) family receptor alpha like (GFRAL) which seems to be exclusively expressed by brainstem neurons at areas of the brain that are key for appetite regulation (Mullican et al., 2017; Tsai et al., 2014; Yang et al., 2017). In rodents, native GDF15 and GDF15 analogues reduce food intake and drive remarkable weight loss (Wang et al., 2023). Current phase 1 data from humans are, however, less encouraging (Benichou et al., 2023).

Plasma concentrations of GDF15 appear to be regulated by many stimuli that initiate cell stress and as part of a wide variety of disease processes including cancer, cardiovascular disease, renal failure, chronic liver disease, anorexia nervosa, and autoimmune diseases, but also physiological changes such as aging, pregnancy, and physical activity (Hüllwegen et al., 2025; Kamper et al., 2024; Plomgaard et al., 2022; Tsai et al., 2018; Wallentin et al., 2013). GDF15 response to nutrient intake shows contradictory results (London et al., 2024; Lu et al., 2023; Martinussen et al., 2021; Patel et al., 2019; Schernthaner‐Reiter et al., 2016; Tsai et al., 2015). Previously, two small studies have reported temporal variations in circulating GDF15: one investigating 14 Asian men and women under fasted conditions (Tsai et al., 2015) and another including five Caucasian men during an overfeeding regimen (Klein et al., 2021). However, both of these controlled conditions are by design artificial and provide limited information about the temporal dynamics of GDF15 in humans under conditions more similar to normal daily life.

Circadian rhythms are entrained by daylight, food intake, environmental temperature, and physical activity (Vetter, 2020) and are controlled by the endogenous master clock located in the hypothalamic suprachiasmatic nuclei and cycling clocks in all peripheral tissues (Mohawk et al., 2012; Takahashi, 2016). Disruption of these rhythms—for example by modern living standards and shift work—is known to be a risk factor for metabolic disorders, including impaired insulin secretion, abnormal glucose tolerance, obesity, and type 2 diabetes (T2D) (Andriessen et al., 2021; Boivin et al., 2022; Hastings et al., 2003).

In order to investigate circadian rhythms, a study needs to be carried out under constant conditions and in the absence of all external time clues (Vetter, 2020). In this study, we investigated whether circulating GDF15 exhibits temporal variation in 22 healthy young men using validated measurement techniques measured over 24 h under conditions approximating normal daily life. Previously published data on plasma glucose, C‐peptide, and glucagon from the same cohort (Zilstorff et al., 2024) were included to explore potential correlations between GDF15 and glucose, glucagon, and β‐cell function.

## 2 MATERIALS AND METHODS

### 2.1 Ethical approval

The study was conducted according to the Helsinki declaration. The local independent ethics committee (protocol number H‐B‐2008‐011) and the Danish Data Protection Agency (journal number 2008‐41‐1821) approved the study. Trial registration ClinicalTrials.gov Identifier: NCT06166368. Registered 12 December 2023. All participants signed an informed written consent before inclusion.

### 2.2 Study design and participants

The study design of this study has been described in detail elsewhere (Sennels et al., 2011; Zilstorff et al., 2024). In brief, a total of 24 healthy males with regular sleep schedules, aged 20–40 years (mean age ± SD: 26 ± 5 years) were included in the original study that was conducted during October and November 2008, on three different study days. In the analyses performed in this manuscript two of the subjects from the original study (Sennels et al., 2011; Zilstorff et al., 2024) were excluded, since they were not fasted at the first blood drawing. After an overnight fast, they were examined for 24 h at the hospital ward with 15 h of wakefulness in normal daylight and 9 h of sleep in the dark from 23:00 h to 8:00 h.

The subjects received three mixed meals: at 9:30 h, 13:00 h, and 19:00 h. Detailed information about the energy distribution of the meals has been reported previously (Zilstorff et al., 2024).

### 2.3 Blood sampling

In brief, every third hour the participants had blood samples drawn (nine time points in total). At the first blood sampling at 9:00 h the participants were fasting from 22:00 h the night before.

Blood samples for GDF15 measurements were drawn in K3EDTA (ethylene diamine tetraacetic acid) plasma tubes (Greiner Bio‐one, Frickenhausen, Germany) and centrifuged immediately. The plasma was stored for −80°C until analysis. Pre‐analytical details regarding blood sample handling for measurements of glucose, C‐peptide, glucagon, and HbA1c have been described previously (Zilstorff et al., 2024).

### 2.4 Biochemical analysis

Plasma GDF15 concentrations were measured with colorimetric enzyme‐linked immunosorbent assays (ELISA) protocols from R&D systems (Catalog Number DFG150, SGD150, PDGD150, R&D systems, USA). The intra‐assay precision (CV%) was reported by the manufacturer to be between 1.8% and 2.8% for three samples of known concentration, tested 20 times each. The inter‐assay precision (CV%) was likewise reported by the manufacturer to be between 4.7% and 6.0% for three samples of known concentration tested 20 times each as well. Details of the biochemical analyses of glucose, C‐peptide, glucagon, and HbA1c have been reported previously (Zilstorff et al., 2024).

### 2.5 Data analysis

Data are presented as mean ± SD. Data were analyzed for a circadian rhythm using the methods for cosinor rhythmometry (Cornelissen, 2014). The 24‐h rhythms were characterized by the rhythm parameters: mesor (rhythm‐adjusted average about which oscillation occurs), amplitude (half the difference between the highest and lowest value of the fitted cosinor curve), and time of peak. Peak times and nadir times are 12 h apart due to the symmetric cosine‐curve model used to analyze the data. Post hoc, individual GDF15 concentration profiles were visualized. Based on this inspection, a subgroup of 14 participants with rhythms peaking in the evening was included in a post hoc exploratory cosinor analysis. In addition, one‐way repeated measures analysis of variance (ANOVA) was performed to test for time effects on circulating GDF15 in the full cohort. Bonferroni‐corrected pairwise comparisons between time points were performed to identify specific differences. For correlation analyses Pearson's correlation analysis was performed.

Statistical analyses were done with R statistical software version 2023.06.0 for Windows. A significance level of 0.05 was used for all hypothesis testing. Earlier published data on plasma concentrations of glucose, C‐peptide, glucagon, and HbA1c were included for correlation analyses and for baseline characteristics (Sennels et al., 2015; Zilstorff et al., 2024).

## 3 RESULTS

### 3.1 Study participants characteristics

The included study participants constituted a homogeneous group of 22 healthy males with normal BMI (22.8 ± 1.5 kg/ m^2^, mean ± SD), and HbA1c (33.9 ± 2.2 mmol/mol) (Table 1). Plasma concentration of GDF15 under fasted conditions was 257.0 ± 58.4 pg/mL (Table 1).

**TABLE 1:** Demographic characteristics and baseline fasting value of GDF15 for the 22 healthy male study participants included in this study. Two participants from the original cohort (*n* = 24) were excluded because they were not fasting at the time of the first blood sample.

	Healthy volunteers (*n* = 22)
Gender	Male (all)
Age (years)	26 ± 5
BMI (kg/m^2^)	22.8 ± 1.5
HbA1c (mmol/mol)	33.9 ± 2.2
HbA1c (%)	5.3 ± 0.2
Fasting GDF15 level (pg/mL)	257.0 ± 58.4

*Note*: Data are presented as mean ± SD. Plasma concentrations of HbA1c have been published previously (Sennels et al., 2015).

Abbreviations: BMI, body mass index; HbA1c, glycated hemoglobin.

### 3.2 GDF15 levels during the study day and night

**FIGURE 1:**
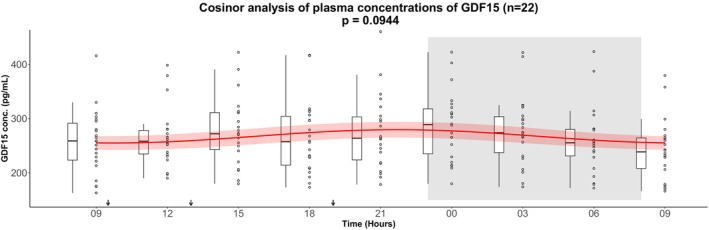
Cosinor analysis of plasma concentrations of GDF15 in 22 healthy males measured over 24 h. After an overnight fast the participants had 15 h of wakefulness (08 h–23 h) followed by a 9‐h sleep opportunity (23 h–08 h) at the hospital ward. The sleep period is indicated with the gray area. The black arrows indicate time points for breakfast (9:30 h), lunch (13:00 h), and dinner (19:00 h). The red curves show the best fitting cosinor curve with confidence bounds for each subject. A boxplot and the full data set are shown. The central box covers the 25th, 50th, and 75th percentile. The whiskers marks 1.5*interquartile range (IQR). Conc., concentration; GDF15, Growth Differentiation Factor 15.

A cosinor analysis of plasma GDF15 concentrations in the full cohort (*n* = 22) did not reveal a statistically significant 24‐hour rhythm (*p* = 0.0944); however, a trend toward rhythmicity was observed, with fluctuations around the rhythm‐adjusted mean (mesor ± SD) 268.5 ± 4.4 pg/mL, a relative amplitude of 4.8%, and an estimated peak time at 21:48 h (Figure 1 and Table 2).

**TABLE 2:** Output from the cosinor analysis of the concentration of GDF15 in 22 healthy males.

Output from GDF15 cosinor analysis (*n* = 22)					
Baseline fasting value (pg/mL)	Mesor (SD) (pg/mL)	Amplitude (SD) (pg/mL)	Peak time	Cosinor *p*	Relative amplitude (%)
257.0 (58.4)	268.5 (4.4)	12.9 (5.9)	21:48	0.0944	4.8

*Note*: GDF15 concentrations were analyzed with cosinor analysis.

Abbreviations: GDF15, Growth Differentiation Factor 15; Amplitude, half the difference between the highest and lowest value of the fitted cosinor curve; Mesor, rhythm adjusted average about which oscillation occurs; Relative amplitude, 100*(amplitude/mesor).

In the full cohort, a one‐way repeated measures ANOVA revealed a significant main effect of time on circulating GDF15 levels (*p* < 0.0001) (see Table 3). Post hoc pairwise comparisons with Bonferroni correction demonstrated significantly higher GDF15 concentrations during the late evening, night, and early morning (24:00 h, 03:00 h, 06:00 h, and 09:00 h next day) compared with earlier daytime values (09:00–18:00 h). The most robust differences were observed between 24:00 h and 09:00 h next day, 03:00 h and 09:00 h next day, 06:00 h and 09:00 h next day, and 24:00 h and 09:00 h next day (see Table S1). Together, these findings indicate a temporal pattern with peak GDF15 levels occurring during the night and early morning hours. However, although significant, the effect size was small, indicating that time accounted for only a modest proportion of the variance in GDF15 levels.

**TABLE 3:** Output from one‐way repeated measures ANOVA of circulating GDF15 levels in 22 healthy young males.

Output from one‐way repeated measures ANOVA					
Effect	Numerator degrees of freedom (DFn)	Denominator degrees of freedom (DFd)	*F*	*p*	Generalized eta‐squared (ges)
Time	4.96	104.1	11.32	<0.001	0.043

*Note*: The table shows the effect of time on circulating GDF15 concentrations.

Abbreviations: DFd, denominator degrees of freedom; DFn, numerator degrees of freedom; F, *F*‐statistic; ges, generalized eta‐squared as a measure of effect size; *p*, *p* value.

**FIGURE 2:**
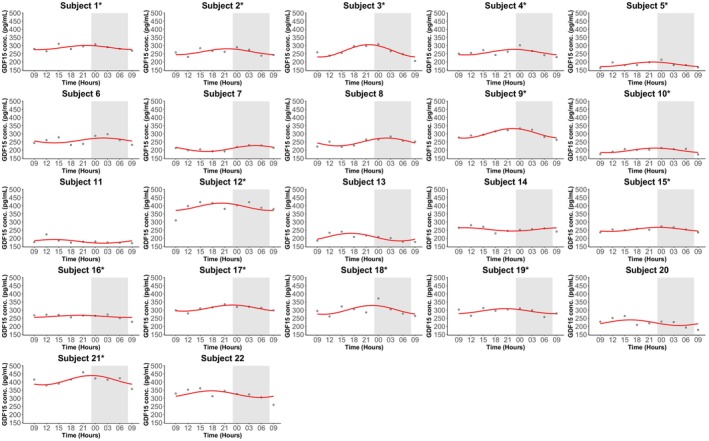
Individual plasma concentrations of GDF15 in 22 healthy males measured over 24 h. After an overnight fast the participants had 15 h of wakefulness (08:00 h–23:00 h) followed by a 9‐h sleep opportunity (23:00 h–08:00 h) at the hospital ward. The sleep period is indicated with the gray area. The black arrows indicate time points for breakfast (9:30 h), lunch (13:00 h), and dinner (19:00 h). The red curves show the best fitting cosinor curve for each subject. The asterisk (*) following the subject number indicate the subjects that were included in the subgroup of 14 subjects whose estimated peak times were within ±3 h of the mean peak time (21:18 h). Conc., concentration; GDF15, Growth Differentiation Factor 15.

**FIGURE 3:**
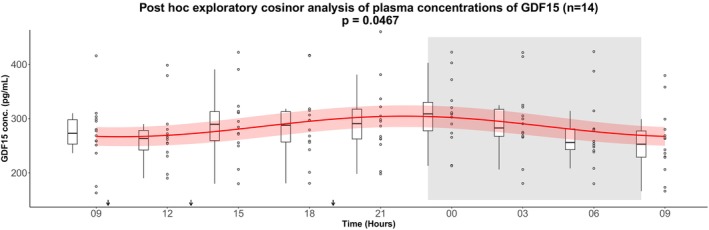
Post hoc exploratory cosinor analysis of plasma concentrations of GDF15 in a subgroup of the subjects including 14 subjects whose estimated peak times were within ±3 h of the mean peak time (21:18 h). GDF15 secretion was measured over 24 h. After an overnight fast the participants had 15 h of wakefulness (08:00 h–23:00 h) followed by a 9‐h sleep opportunity (23:00 h–08:00 h) at the hospital ward. The sleep period is indicated with the gray area. The black arrows indicate time points for breakfast (9:30 h), lunch (13:00 h), and dinner (19:00 h). The red curves show the best fitting cosinor curve with confidence bounds for each subject. A boxplot and the full data set are shown. The central box covers the 25th, 50th, and 75th percentile. The whiskers marks 1.5*interquartile range (IQR). Conc., concentration; GDF15, Growth Differentiation Factor 15.

**FIGURE 4:**
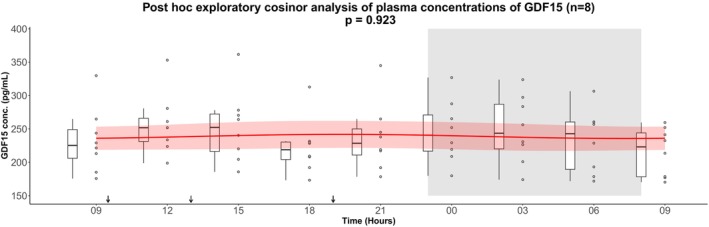
Post hoc exploratory cosinor analysis of plasma concentrations of GDF15 from the eight subjects whose estimated peak times were > ±3 h from the mean peak time (21:18 h). GDF15 secretion was measured over 24 h. After an overnight fast the participants had 15 hours of wakefulness (08:00 h–23:00 h) followed by a 9‐h sleep opportunity (23:00 h–08:00 h) at the hospital ward. The sleep period is indicated with the gray area. The black arrows indicate time points for breakfast (9:30 h), lunch (13:00 h) and dinner (19:00 h). The red curves show the best fitting cosinor curve with confidence bounds for each subject. A boxplot and the full data set are shown. The central box covers the 25th, 50th, and 75th percentile. The whiskers marks 1.5*interquartile range (IQR). Conc., concentration; GDF15, Growth Differentiation Factor 15.

As a post hoc exploratory analysis, we examined individual GDF15 plasma concentration profiles. In four of the 22 participants, cosinor analysis indicated a significant 24‐h rhythm (Figure 2 and Table S2). Visual inspection suggested that most participants exhibited fluctuations with peaks in the evening or early night. Based on this observation, we performed a post hoc exploratory cosinor analysis in a subgroup of 14 subjects whose estimated peak times were within ±3 hours of the mean peak time from the entire cohort (21:18 h). In this subgroup, a significant 24‐hour rhythm was detected (*p* = 0.0467), with concentrations fluctuating around the rhythm‐adjusted mean (mesor ± SD) 285.5 ± 5.6 pg/mL, a relative amplitude of 6.6%, and a peak time at 22:00 h (Figure 3 and Table 4). Conversely, a cosinor analysis of the remaining eight subjects (peak times > ± 3 h from the mean peak time) did not show a significant rhythm, and visual inspection indicated heterogeneous patterns inconsistent with a clear 24‐h rhythm (Figure 4).

**TABLE 4:** Output from the post hoc exploratory cosinor analysis of plasma concentrations of GDF15 in a subgroup of the subjects including 14 subjects whose estimated peak times were within ±3 h of the mean peak time (21:18 h).

Output from GDF15 post hoc exploratory cosinor analysis (*n* = 14)					
Baseline fasting value (pg/mL)	Mesor (SD) (pg/mL)	Amplitude (SD) (pg/mL)	Peak time	Cosinor *p*	Relative amplitude (%)
270.8 (58.4)	285.5 (5.6)	18.9 (7.6)	22:00	0.0467	6.6

Abbreviations: Amplitude, half the difference between the highest and lowest value of the fitted cosinor curve; GDF15, Growth Differentiation Factor 15; Mesor, rhythm adjusted average about which oscillation occurs; Relative amplitude, 100*(amplitude/mesor).

### 3.3 Determinants of plasma GDF15 during a day

**FIGURE 5:**
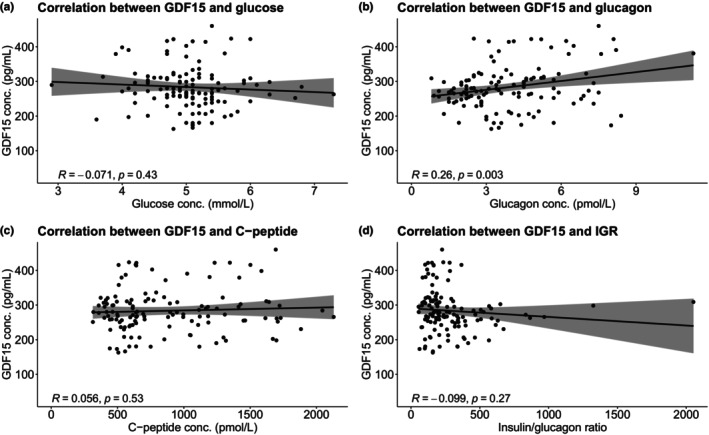
Pearson's correlation analyses between (a) GDF15 and glucose, (b) GDF15 and glucagon, (c) GDF15 and C‐peptide, and (d) GDF15 and IGR for the subgroup including the 14 subjects whose estimated peak times were within ±3 h of the mean peak time (21:18 h). IGR is calculated as the ratio between plasma concentration of C‐peptide and plasma concentration of glucagon. Conc., concentration; GDF15, Growth Differentiation Factor 15; IGR, insulin/glucagon ratio.

To investigate if plasma concentrations of GDF15 depended on plasma concentrations of glucose, glucagon, C‐peptide or the ratio between C‐peptide and glucagon (representing insulin/glucagon ratio (IGR)) we performed correlation analyses for the subgroup including the 14 subjects whose estimated peak times were within ±3 h of the mean peak time (21:18 h). We did not observe statistically significant correlations between plasma GDF15 and glucose (*R* = −0.071, *p* = 0.43), between GDF15 and C‐peptide (R = 0.056, *p* = 0.53), or between GDF15 and IGR (*R* = −0.099, *p* = 0.27). We did, however, see a weak positive but statistically significant correlation between plasma GDF15 and plasma glucagon (*R* = 0.26, *p* = 0.003) (Figure 5).

## 4 DISCUSSION

Although the cosinor analysis in the full cohort did not reveal a statistically significant 24‐h rhythm, testing the same data by one‐way repeated measures ANOVA indicated a significant main effect of time on circulating GDF15 in the full cohort of 22 healthy young men. Post hoc comparisons suggested that GDF15 concentrations were higher during the late evening, night, and early morning compared with daytime values. This apparent discrepancy can be explained by methodological differences: the cosinor analysis tests whether the data follow a symmetric sinusoidal rhythm, whereas the ANOVA evaluates whether differences exist between individual time points without assuming a specific rhythm. Thus, while the outcome of the cosinor analysis does not support a strict 24‐h rhythm, the ANOVA findings suggest that GDF15 levels vary modestly during 24 h, particularly with elevations at night. Exploratory post hoc cosinor analyses suggested that a subgroup of subjects (*n* = 14) displayed modest rhythmic fluctuations with an evening peak at 22:00 h with a relative amplitude of 6.6%. However, these fluctuations were considerably smaller than those observed for other hormones in the same cohort, such as C‐peptide (40.5%) and glucagon (20.5%) (Zilstorff et al., 2024). The modest daily variations in GDF15 are small compared with changes observed under physiological or pathological conditions. For instance, plasma GDF15 concentrations increase several‐fold with advanced age, malignancy, or pregnancy (Hüllwegen et al., 2025). Although GDF15 may fluctuate across the day in some individuals, the clinical relevance of these modest variations remains uncertain. Moreover, the subgroup analysis was defined post hoc, underlining the need for replication in prospectively designed studies.

Correlation analyses indicated a weak positive association between plasma GDF15 and glucagon, in line with previous findings (Plomgaard et al., 2022; Richter et al., 2024), whereas no significant associations were observed with glucose, C‐peptide, or insulin/glucagon ratio. These data are consistent with earlier studies suggesting that food intake and fasting do not acutely alter circulating GDF15 (Martinussen et al., 2021; Patel et al., 2019; Tsai et al., 2015).

Several limitations should be acknowledged. First, the study sample was modest, no a priori power calculation was performed, and the primary cosinor analysis in the full cohort was not statistically significant. Second, the post hoc subgroup cosinor analysis was exploratory, limiting the strength of conclusions. Third, to establish a rhythmic pattern it is most ideal to repeat the measurements on at least three separate days, which unfortunately was not done in this study. Fourth, sleep quality was not objectively monitored by EEG or polysomnography (PSG), which would have allowed for a more precise evaluation of the relationship between sleep and GDF15 dynamics. Fifth, the amplitude of the observed fluctuations was small compared with the fluctuations of other metabolic markers and compared to the levels of GDF15 observed, for example, in pregnancy and cancer. Finally, seasonal variation may influence circadian rhythmicity, and this potential seasonal effect should be taken into account in future research.

Future studies should include larger, prospectively powered cohorts, objective sleep monitoring, and more frequent blood sampling over at least 3 days to better characterize the temporal profile of GDF15.

## 5 CONCLUSION

In conclusion, our findings do not provide definitive evidence for a 24‐h rhythm of circulating GDF15 in healthy young men, but a significant time effect was observed. Exploratory analyses suggest that some individuals may display modest temporal variations over 24 h, but the fluctuations are small, and require confirmation in larger, prospectively designed studies. The clinical implications of potential temporal variation in GDF15 levels therefore remain to be established.

## AUTHOR CONTRIBUTIONS

NJWA and MMR conceived the idea of evaluating GDF15 levels during circadian rhythm. HJ and HS conducted the clinical experiments and provided plasma samples for analyses. MMR, JH, HJ, HS, RK and CC provided intellectual support. DBZ wrote the first draft of the manuscript. All authors revised and accepted the final version of the manuscript.

## FUNDING INFORMATION

Associate Prof. Nicolai J. Wewer Albrechtsen is supported by NNF Excellence Emerging Investigator Grant – Endocrinology and Metabolism (Application No. NNF19OC0055001), EFSD Future Leader Award (NNF21SA0072746) and DFF Sapere Aude (1052‐00003B). NNF Center for Protein Research is supported financially by the NNF (grant agreement NNF14CC0001). However, the work reported in this manuscript was not directly funded or sponsored.

## CONFLICT OF INTEREST STATEMENT

NJWA has received funding from and served on scientific advisory panels and/or speakers' bureaus for Boehringer Ingelheim, MSD/MERCK, Roche, Novo Nordisk and Mercodia. RK is a senior scientist at Novo Nordisk A/S. Novo Nordisk A/S has not had any influence on the design, analysis, interpretation, or writing of this article. The remaining authors have no conflicts of interest to declare.

## CONSENT

Not applicable.

## Supporting information


Tables S1–S2.

## Data Availability

The data that support the findings of this study are available from the corresponding author upon request. The codes used to process and analyze the data in this study are also available from the corresponding author upon request.
