# Supplementary material for: Temporal variation in circulating GDF15 over 24 h in healthy young males
Source: Physiol Rep. 2025 Nov 24;13(22):e70672. doi: 10.14814/phy2.70672 (PMC12641278; doi:10.14814/phy2.70672)
Supplement: Supplementary file 1 — Tables S1–S2. [file PHY2-13-e70672-s001.docx]

**Supplementary**

| **Comparison** | ***p*-value (raw)** | ***p*-value (Corrected)** |
| --- | --- | --- |
| 09h vs 12h | 0.248 | 1 |
| 09h vs 15h | <0.01 | 0.062 |
| 09h vs 18h | 0.2 | 1 |
| 09h vs 21h | <0.01 | 0.133 |
| 09h vs 24h | <0.0001 | <0.01 |
| 09h vs 03h | <0.01 | 0.092 |
| 09h vs 06h | 0.718 | 1 |
| 09h vs 09h (day 2) | <0.01 | 1 |
| 12h vs 15h | <0.05 | 1 |
| 12h vs 18h | 0.976 | 1 |
| 12h vs 21h | 0.219 | 1 |
| 12h vs 24h | <0.05 | 0.472 |
| 12h vs 03h | 0.057 | 1 |
| 12h vs 06h | 0.337 | 1 |
| 12h vs 09h (day 2) | 0.001 | <0.05 |
| 15h vs 18h | <0.05 | 1 |
| 15h vs 21h | 0.567 | 1 |
| 15h vs 24h | 0.138 | 1 |
| 15h vs 03h | 0.963 | 1 |
| 15h vs 06h | <0.01 | 0.293 |
| 15h vs 09h (day 2) | <0.0001 | <0.001 |
| 18h vs 21h | <0.05 | 1 |
| 18h vs 24h | <0.0001 | <0.01 |
| 18h vs 03h | <0.05 | 0.432 |
| 18h vs 06h | 0.236 | 1 |
| 18h vs 09h (day 2) | <0.001 | <0.05 |
| 21h vs 24h | <0.05 | 1 |
| 21h vs 03h | 0.503 | 1 |
| 21h vs 06h | <0.01 | 0.231 |
| 21h vs 09h (day 2) | <0.0001 | <0.01 |
| 24h vs 03h | 0.05 | 1 |
| 24h vs 06h | <0.0001 | <0.01 |
| 24h vs 09h (day 2) | <0.0001 | <0.0001 |
| 03h vs 06h | <0.0001 | <0.001 |
| 03h vs 09h (day 2) | <0.0001 | <0.0001 |
| 06h vs 09h (day 2) | <0.0001 | <0.01 |

**Supplementary table 1. Post hoc pairwise comparisons of circulating GDF15 concentrations between time points in the full cohort (n = 22).**Results are *p*-values from raw pairwise paired t-tests and p-values with Bonferroni correction for multiple comparisons. Time points are expressed as clock time (h).

|  | | **Output from individual cosinor analysis** | | | |
| --- | --- | --- | --- | --- | --- |
| **Subject number** | **Baseline fasting value**  **(pg/mL)** | **Mesor  (pg/mL)** | **Amplitude**  **(pg/mL)** | **Peak, clock time** | **Cosinor *p*** |
| **1*** | 279.9 | 288.0 | 13.0 | 21:49 | 0.217 |
| **2*** | 258.4 | 262.9 | 17.7 | 21:38 | 0.170 |
| **3*** | 259 | 268.1 | 37.8 | 21:38 | <0.05 |
| **4*** | 251.1 | 260.0 | 16.8 | 22:35 | 0.260 |
| **5*** | 162.9 | 185.8 | 13.7 | 21:49 | 0.176 |
| **6** | 243.7 | 259.4 | 15.1 | 02:00 | 0.524 |
| **7** | 213.1 | 209.5 | 18.2 | 04:35 | <0.05 |
| **8** | 221.5 | 250.1 | 24.1 | 02:00 | 0.0551 |
| **9*** | 277.2 | 303.1 | 29.3 | 22:12 | <0.05 |
| **10*** | 174.8 | 198.6 | 13.5 | 22:29 | 0.0998 |
| **11** | 175.7 | 181.0 | 11.4 | 13:52 | 0.434 |
| **12*** | 309.8 | 393.4 | 22.3 | 20:13 | 0.420 |
| **13** | 185.1 | 207.0 | 23.5 | 17:16 | 0.0800 |
| **14** | 264.8 | 254.6 | 10.2 | 10:13 | 0.411 |
| **15*** | 236.2 | 254.6 | 12.6 | 23:19 | 0.0725 |
| **16*** | 268.4 | 262.1 | 6.5 | 19:58 | 0.679 |
| **17*** | 298.7 | 312.7 | 18.8 | 22:55 | <0.05 |
| **18*** | 295.1 | 301.9 | 27.2 | 22:28 | 0.232 |
| **19*** | 303.5 | 294.0 | 15.0 | 20:26 | 0.279 |
| **20** | 229.1 | 223.0 | 17.3 | 16:21 | 0.480 |
| **21*** | 415.7 | 411.6 | 28.8 | 23:04 | 0.0888 |
| **22** | 329.8 | 325.8 | 20.3 | 17:46 | 0.426 |

**Supplementary Table 2.** Individual cosinor analyses of plasma concentrations of GDF15 in 22 healthy males measured over 24 hours. Mesor: rhythm adjusted average about which oscillation occurs, Amplitude: half the difference between the highest and lowest value of the fitted cosinor curve. The asterix(*) following the subject number indicate the subjects that were included in the subgroup of 14 subjects whose estimated peak times were within ±3 hours of the mean peak time (21:18 h).
